# *In situ* regeneration of bioactive coatings enabled by an evolved *Staphylococcus aureus* sortase A

**DOI:** 10.1038/ncomms11140

**Published:** 2016-04-13

**Authors:** Hyun Ok Ham, Zheng Qu, Carolyn A. Haller, Brent M. Dorr, Erbin Dai, Wookhyun Kim, David R. Liu, Elliot L. Chaikof

**Affiliations:** 1 Department of Surgery, Beth Israel Deaconess Medical Center, Harvard Medical School, Boston, Massachusetts 02215, USA; 2 Coulter Department of Biomedical Engineering, Petit Institute for Bioengineering and Bioscience, Georgia Institute of Technology, Atlanta, Georgia 30332, USA; 3 Howard Hughes Medical Institute, Department of Chemistry and Chemical Biology, Harvard University, Cambridge, Massachusetts 02138, USA; 4 Wyss Institute of Biologically Inspired Engineering, Harvard University, Boston, Massachusetts 02115, USA

## Abstract

Surface immobilization of bioactive molecules is a central paradigm in the design of implantable devices and biosensors with improved clinical performance capabilities. However, *in vivo* degradation or denaturation of surface constituents often limits the long-term performance of bioactive films. Here we demonstrate the capacity to repeatedly regenerate a covalently immobilized monomolecular thin film of bioactive molecules through a two-step stripping and recharging cycle. Reversible transpeptidation by a laboratory evolved *Staphylococcus aureus* sortase A (eSrtA) enabled the rapid immobilization of an anti-thrombogenic film in the presence of whole blood and permitted multiple cycles of film regeneration *in vitro* that preserved its biological activity. Moreover, eSrtA transpeptidation facilitated surface re-engineering of medical devices *in situ* after *in vivo* implantation through removal and restoration film constituents. These studies establish a rapid, orthogonal and reversible biochemical scheme to regenerate selective molecular constituents with the potential to extend the lifetime of bioactive films.

## Abstract (web summary)

Bioactive coatings offer a strategy to modulate host response to implants, but their translation to the clinic is hampered by their fast *in vivo* degradation. Here, the authors use an engineered bacterial protein to regenerate an anti-thrombogenic film *in vitro* and *in situ* after device implantation.

## Graphical abstract

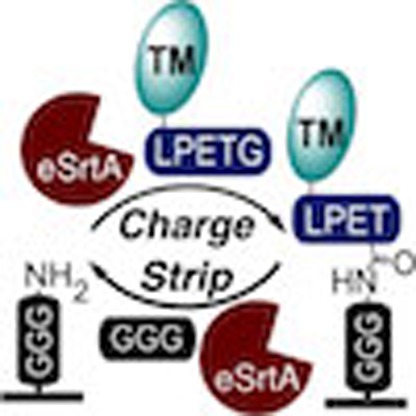

Medical devices in blood-contacting applications, such as stents, heart valves, ventricular assist devices and extracorporeal support systems, as well as vascular grafts and access catheters are prone to failure due to maladaptive host responses at the blood–material interface^1^,^2^,^3^,^4^,^5^,^6^. Immobilization of bioactive molecules and drug eluting thin films on implantable devices have yielded promising combination products that abrogate thrombotic cascades and detrimental inflammatory reactions^7^,^8^,^9^, enhance device integration and local tissue repair^10^ and inhibit microbial colonization^11^,^12^. However, the clinical translation of these strategies remains constrained by the limited therapeutic duration afforded by a finite reservoir of bioactive agents, as well as by the degradation or denaturation of surface components by oxidation, hydrolysis and proteolysis when exposed over time to a physiological environment^11^,^13^,^14^,^15^. Efforts to improve the stability and activity of thin film constituents for both biomedical and biotechnological applications have included attempts to judiciously alter surface properties, such as hydrophilicity, charge, topography^16^,^17^,^18^ and immobilization chemistry^19^,^20^,^21^,^22^, as well as through rational and evolutionary protein engineering^23^,^24^. Despite recent progress in these areas, a surface coating for implantable devices that reliably retains biological activity over extended clinically relevant timescales has not been developed.

In principle, enzyme ligation offers an opportunity for catalysing reversible bond-forming reactions that could enable the molecular regeneration of bioactive thin films *in situ*. *Staphylococcus aureus* sortase A (SrtA) catalyses the covalent transpeptidation of a C-terminal ‘sorting motif' LPXTG to N-terminal oligoglycine (for example, GGG) nucleophiles through an acyl-enzyme complex forming an LPXT-GGG bond^25^,^26^. Because of its synthetic simplicity and the very limited occurrence of the LPXTG motif in native proteins, SrtA-catalysed transpeptidation has been broadly applied in protein purification, labelling and immobilization onto solid supports^27^,^28^,^29^,^30^,^31^. However, the low catalytic activity and substrate affinity of wild-type (WT) SrtA necessitates high molar excess of the enzyme and long incubation times to approach reaction completion, limiting its effectiveness and applicability. An evolved SrtA mutant (eSrtA) has recently been generated that exhibits 120-fold higher LPETG-ligation activity than the WT enzyme^30^,^32^. This enzyme suggested the possibility of multiple rapidly catalysed cycles of removal and reversible assembly of bioactive molecular films onto oligoglycine-modified surfaces both *in vitro* and *in vivo*. Indeed, we observed that eSrtA transpeptidation was reversible, enabling multiple cycles of peptide bond formation and cleavage, and, thereby, facilitating film regeneration in the presence of whole blood *in vitro* and *in vivo*. These studies establish a rapid, orthogonal and reversible scheme, which can be used to regenerate selective molecular constituents with the potential to extend the lifetime of bioactive films, or likewise, as a method to load and release any of a number of material bound constituents for controlled drug loading and delivery, or as a strategy for material dissolution.

## Results

### An evolved sortase catalyses rechargeable molecular assembly

**Figure 1:**
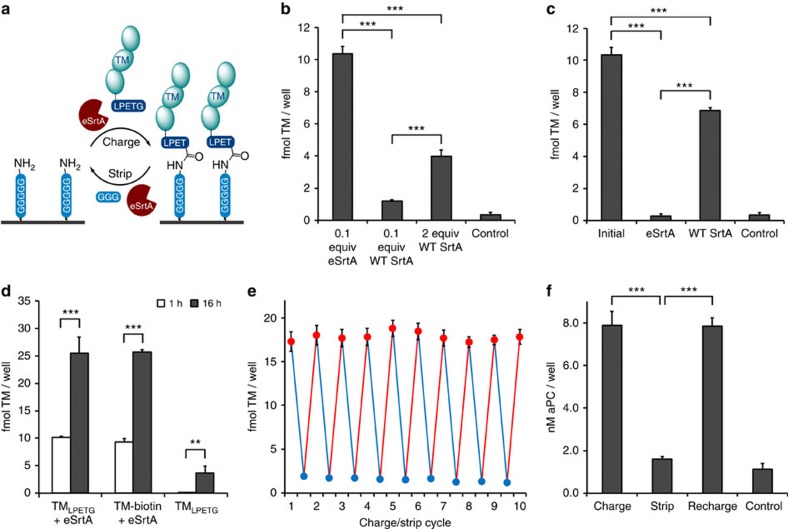
Sortase-catalysed rechargeable surface assembly. (**a**) Two-step ‘rechargeable' surface assembly cycle initiated by sortase-catalysed charging of LPETG-tagged biomolecules on pentaglycine-modified surfaces, followed by sortase-catalysed stripping to regenerate pentaglycine anchor sites for additional surface reaction cycles. (**b**) Immobilization of 1 μM TM_LPETG_ on pentaglycine-coated microwells using 0.1 molar equivalents evolved sortase (eSrtA), 0.1 and 2 molar equivalents WT SrtA, or no sortase as a negative control. (**c**) Following immobilization of 1 μM TM_LPETG_ on pentaglycine-coated microwells using 0.1 molar equivalents eSrtA, removal of bound TM was carried out using 20 μM of either evolved eSrtA or WT SrtA with 1 mM triglycine. (**d**) Sortase-catalysed binding of TM_LPETG_ on pentaglycine-modified model surfaces following 1 h and 16 h reactions were compared with the binding of biotinylated TM (TM-biotin) directly on streptavidin-coated microwells. In parallel, TM_LPETG_ was incubated in microwells without sortase as a negative control. (**e**) Rapid repetitive recharging of TM_LPETG_ on pentaglycine surfaces by eSrtA. Sequential eSrtA-catalysed charging (red) and stripping (blue) cycles of TM_LPETG_ on pentaglycine surfaces (*n*=4). (**f**) Co-factor activity of immobilized TM to catalyse production of activated protein C (aPC) following the initial assembly of TM_LPETG_, stripping by eSrtA, and regeneration of TM_LPETG_ by eSrtA. **P<0.01, ***P<0.001 (Student's *t*-test), error bars denote s.d. (*n*⩾3).

**Figure 2:**
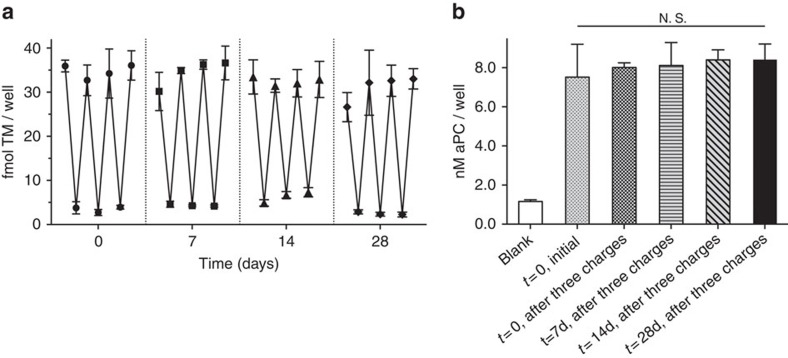
Repetitive surface assembly and removal over 28 days *in vitro*. (**a**) Sequential eSrtA-catalysed charging and stripping of TM_LPETG_ performed at 7-day intervals over a 28-day incubation. (**b**) aPC activity was confirmed on initial surfaces and after three strip/charge events performed on days 7, 14 and 28. N.S. (not significant), error bars denote s.d. (*n*⩾3).

We have extensively characterized the ability of thrombomodulin (TM) to limit tissue factor induced thrombin production under venous and arterial flow conditions using both experimental and computational models^31^,^33^,^34^,^35^,^36^,^37^,^38^. Surfaces functionalized with TM, a major physiological inhibitor of thrombin generation^39^,^40^, exhibit reduced thrombogenicity *in vitro* and *in vivo*, thereby, supporting its potential utility in blood-contacting devices^31^,^37^,^41^,^42^,^43^,^44^,^45^,^46^,^47^,^48^,^49^. To demonstrate the ability to reversibly assemble TM on oligoglycine surfaces in a site-specific manner (Fig. 1a), we first generated a recombinant human TM fragment containing a C-terminal LPETG motif (TM_LPETG_). WT sortase (WT SrtA) and an eSrtA produced by directed evolution using a yeast display system were generated, as previously reported^32^. Model pentaglycine surfaces, which match the natural SrtA substrate on the *S. aureus* cell wall^50^ were constructed by immobilizing a biotinylated pentaglycine peptide (NH_2_-GGGGGK-biotin) on surfaces pre-coated with streptavidin. Surface immobilization of TM_LPETG_ using eSrtA yielded a ∼10-fold higher surface density than that attained in the presence of an equivalent concentration of WT SrtA. Further, despite a 20-fold increase in WT SrtA, only a modest increase in TM surface density was observed (Fig. 1b). Near-complete removal of immobilized TM films could be achieved with eSrtA in 30 min, whereas WT SrtA was substantially less efficient under identical conditions (Fig. 1c). In this system, the binding kinetics of TM_LPETG_ by eSrtA roughly approximated those of biotin-streptavidin (Fig. 1d). As illustrated by 10 repetitive stripping and charging cycles using eSrtA, rapid and reproducible regeneration of surface-bound TM films proved feasible with preservation of surface catalytic activity and sustained production of activated protein C (aPC) (Fig. 1e,f). TM_LPETG_-pentaglycine surfaces were incubated for up to 28 days in Tris-buffered saline (TBS) at 37 °C and subjected to three cycles of stripping and recharging at 7-day intervals. We observed no loss in efficiency of eSrtA stripping or recharging as characterized by TM ELISA and TM co-factor activity over this 4-week period (Fig. 2a,b). These results confirmed that the enhanced catalytic activity of eSrtA is a critical enabling step in the application of a two-step strip/recharge scheme for rapid and repetitive regeneration of biologically active molecular films.

### Repetitive molecular recharging can be performed in blood

**Figure 3:**
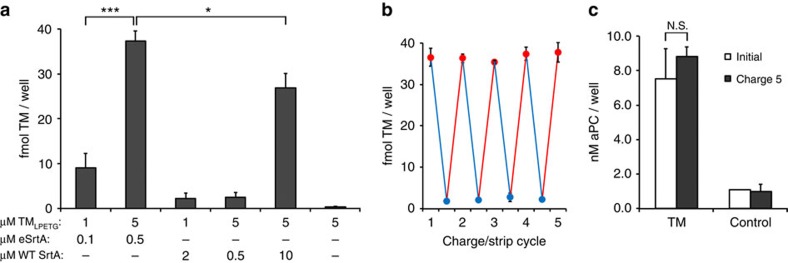
Repetitive surface recharging in the presence of whole blood. (**a**) Direct sortase-catalysed assembly of TM_LPETG_ in 50% v/v heparinized whole blood (20 U heparin per ml of blood) at 37 °C for 1 h without additional calcium. Evolved and WT sortases were tested at two different TM_LPETG_ concentrations as well as TM_LPETG_/sortase ratios. (**b**) Sequential eSrtA-catalysed charging (red) and stripping (blue) cycles of TM_LPETG_ performed on model pentaglycine surfaces in 50% v/v heparinized whole blood (20 U heparin per ml of blood) at 37 °C for 1 h without additional calcium. (**c**) TM co-factor activity (nM aPC/well) was characterized on initial surfaces and after the fifth generation of charging. Blank wells were included as control. N.S. (not significant), *P<0.05, ***P<0.001 (Student's *t*-test), error bars denote s.d. (*n*⩾3).

Because of the rarity of LPETG and GGG motifs in native proteins, we postulated that the two-step strip/recharge reaction mediated by eSrtA would provide a feasible *in vivo* strategy to selectively regenerate molecular constituents on or within medical implants *in situ*. As proof-of-concept, we demonstrated the ability to immobilize TM_LPETG_ on pentaglycine surfaces in 50% v/v whole blood at 37 °C. We observed that eSrtA was capable of efficiently functionalizing model surfaces greater than 20-fold more effectively than WT SrtA (Fig. 3a). In addition, eSrtA was capable of functionalizing surfaces in whole blood at levels equivalent to that achieved in defined reaction buffer with only a five fold increased concentration (0.1 equivalents versus 0.5 equivalents). We performed repeated cycles of stripping/recharging in 50% v/v whole blood and observed both efficient removal and regeneration of TM. Significantly, aPC generation after five rounds of charging was equivalent to that observed on our initial model surface (Fig. 3b,c). These results underscore the enhanced activity of eSrtA as an important enabling step towards *in situ* assembly of molecular films at the blood-contacting interface of medical implants.

### Sortase mediates molecular assembly and disassembly *in vivo*

**Figure 4:**
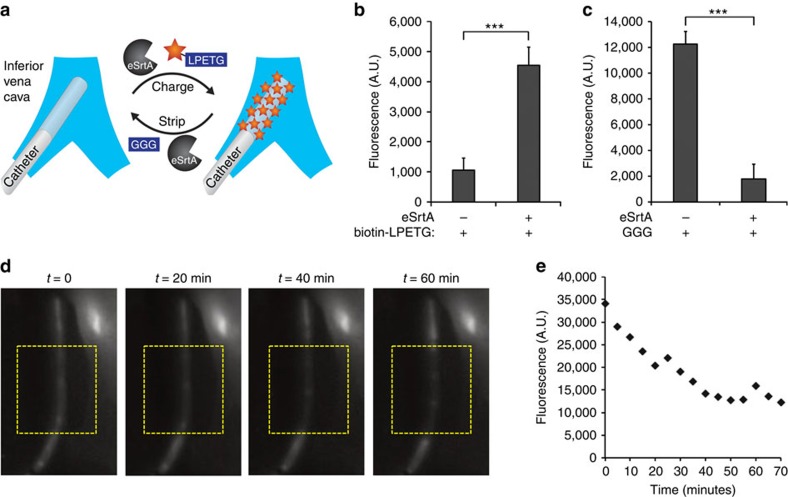
Sortase-catalysed reversible surface assembly *in vivo*. (**a**) Reversible assembly of LPETG-labelled probes (biotin-LPETG or Alexa Fluor 750-LPETG) on pentaglycine-modified catheters deployed *in vivo* was achieved by intravenous delivery of eSrtA and triglycine peptide. (**b**) *In situ* eSrtA-catalysed immobilization of LPETG-tagged biotin (biotin-LPETG) probes on pentaglycine-modified catheters in the mouse inferior vena cava, 1 h after intravenous delivery of biotin-LPETG and eSrtA, catheters were explanted and incubated with Cy3-streptavidin to assess biotin surface density. (**c**) *In situ* eSrtA-catalysed removal of LPETG-tagged biotin probes from pentaglycine-modified catheters deployed in the mouse inferior vena cava, 1 h after intravenous delivery of triglycine (GGG) and eSrtA, catheters were explanted and incubated with Cy3-streptavidin to assess biotin surface density. (**d**) *In situ* removal of LPETG-tagged near-IR probes from pentaglycine-modified PU catheters deployed in mouse vena cava. Representative real-time fluorescent imaging of a pentaglycine-modified polyurethane catheter functionalized with LPETG-labelled Alexa Fluor 750 deployed in the mouse vena cava and stripped by intravenous delivery of eSrtA and triglycine. Catheters modified with pentaglycine peptide and conjugated with LPETG-labelled Alexa Fluor 750 were stripped by IV delivery of triglycine (400 μg) and eSrtA (700 μg). The near-IR fluorescent signal from the modified catheter was monitored using the Maestro multi-spectral fluorescence imaging system in real time over 70 min and (**e**) quantitative image analysis was performed to evaluate the fluorescent signal intensity of the modified catheters. ***P<0.001 (Student's *t*-test), error bars denote s.d. (*n*⩾3).

Polyurethane venous access catheters are widely used, but continue to be limited by a risk of thrombosis and infection, despite attempts to limit these risks by surface immobilization of bioactive molecular films^51^,^52^,^53^,^54^,^55^. As venous catheters are available in sizes compatible for small animal studies, they provide a convenient model system to assess stripping and recharging of surface-bound biologically active molecules *in vivo*. Initial investigations focused on the application of eSrtA transpeptidation to immobilize and remove LPETG-tagged probes on pentaglycine-modified polyurethane catheters deployed in the mouse inferior vena cava. Catheters were modified with cyclooctyne motifs using a sequential scheme (Supplementary Fig. 1), as previously reported^31^,^37^, and azide reactivity was confirmed using rhodamine azide probes (Supplementary Fig. 2). An azide-tagged pentaglycine peptide (NH_2_-GGGGG-N_3_) was used to generate pentaglycine motifs on catheters. Reaction parameters for eSrtA-mediated attachment and removal of a biotin-LPETG probe on catheters were first determined under *in vitro* conditions (Supplementary Figs 3–5). Using 6–8-week-old C57BL/6 mice, pentaglycine-modified catheters were inserted via the femoral vein into the inferior vena cava, followed by intravenous administration of eSrtA with either biotin-LPETG or triglycine (GGG) to charge or strip the catheter surface, respectively (Fig. 4a). Analysis of explanted catheters confirmed the selective immobilization or removal of biotin-LPETG probes within 1 h (Fig. 4b,c and Supplementary Figs 6–9). In a parallel set of experiments using catheters bearing Alexa Fluor 750-LPETG, real-time fluorescent imaging confirmed that the surface-bound near-IR probe was nearly completely removed within 1 h after intravenous administration of eSrtA and GGG (Fig. 4d,e).

**Figure 5:**
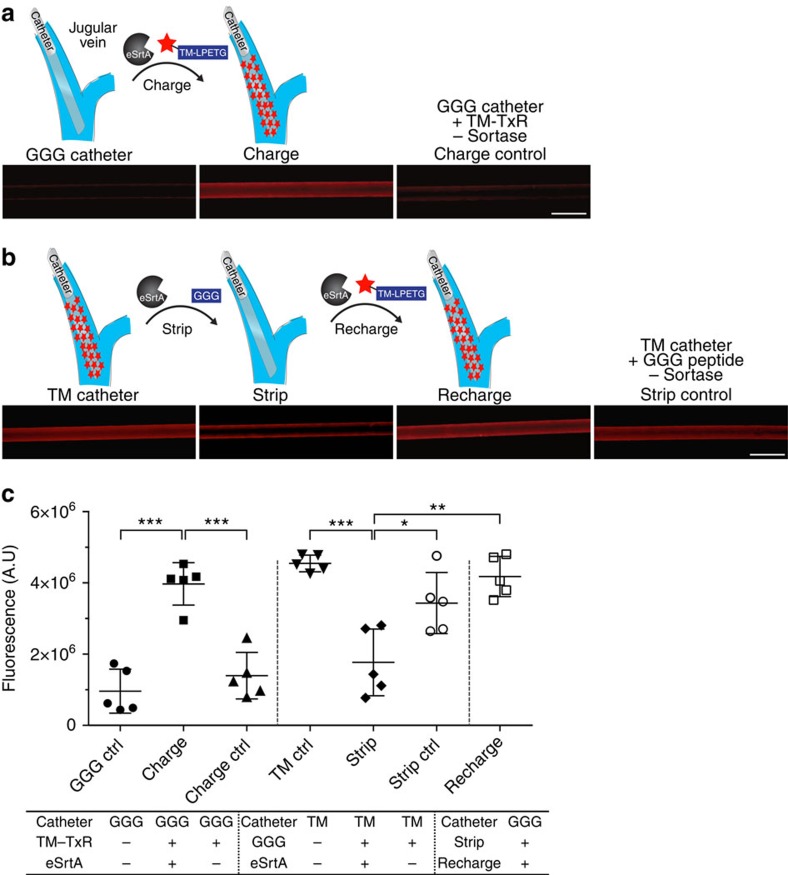
*In situ* molecular recharging of venous catheters after *in vivo* deployment. (**a**) To characterize *in situ* charging of a chronically implanted venous catheter, pentaglycine-modified polyurethane catheters were initially deployed in the rat jugular vein for 7 days. Texas Red-labelled TM_LPETG_ with or without eSrtA was systemically administered by intravenous injection via the dorsal penile vein. Catheters were removed 1 h later and imaged using fluorescence microscopy. Representative images are shown (scale bar, 1 mm). (**b**) To characterize *in situ* stripping, pentaglycine catheters linked to Texas Red-TM were deployed in the rat jugular vein for 7 days. GGG with or without eSrtA was delivered intravenously and 1 h later catheters were removed and imaged. To examine *in situ* recharging, Texas Red-TM catheters were first stripped by intravenous administration of GGG and eSrtA and 24 h later, Texas Red-TM_LPETG_ and eSrtA was administered intravenously. Catheters were removed 1 h later for imaging. Representative images are shown (scale bar, 1 mm). (**c**) Catheter fluorescence intensity was quantified using ImageJ and plotted as. *P<0.05, **P<0.01, ***P<0.001 (Student's *t*-test), error bars denote s.d. (*n*⩾3). A.U., arbitrary units.

To determine whether nonspecific adsorption of plasma proteins or other blood components limits surface accessibility to systemically delivered eSrtA, catheters were deployed in the jugular vein for 7 days using a rat model. Pentaglycine-derivatized polyurethane catheters were inserted and after 1 week, Texas Red-labelled TM_LPETG_ was administered intravenously with or without eSrtA. Catheters were explanted 1 h later and TM surface conjugation was confirmed using fluorescence microscopy (Fig. 5a and Supplementary Fig. 10). *In situ* stripping of Texas Red-TM_LPETG_ and recharging efficiency was examined in a similar manner. Specifically, pentaglycine catheters functionalized with Texas Red-TM_LPETG_ were deployed within the jugular vein for 7 days. Catheters were removed 1 h after intravenous administration of GGG with or without eSrtA and stripping verified by fluorescence imaging (Fig. 5b and Supplementary Fig. 10). *In situ* recharging was assessed by first initiating the *in vivo* removal of TM_LPETG_ from catheter surfaces after deployment within the rat jugular vein for 7 days, as detailed above, followed 24 h later by intravenous administration of Texas Red-TM_LPETG_ with or without eSrtA. Significantly, TM surfaces could be charged, stripped and once again recharged with TM to levels observed before catheter implantation (Fig. 5b,c and Supplementary Fig. 10).

## Discussion

Current techniques to covalently modify surfaces with bioactive compounds have largely involved bioconjugation schemes that link nucleophilic motifs such as amines, thiols and hydroxyls to respective partner electrophiles^56^. The abundant presentation of these motifs in the complex chemical landscape of biological systems reduces the efficiency of targeting payloads to regenerate device reservoirs or surfaces. Recent efforts to modify blood-contacting surfaces with bioactive molecules have led to the application of a variety of bio-orthogonal coupling chemistries, notably Staudinger ligation and native chemical ligation, as well as click cycloaddition and intein-mediated splicing^37^,^57^,^58^,^59^,^60^,^61^,^62^. However, these irreversible bond-forming approaches prohibit the *in situ* removal and regeneration of surface assemblies. Non-covalent strategies for recharging bioactive surfaces have been proposed through the use of metal-affinity^63^, N-halamine structures^64^, disulfide bonds^65^ as well as through biotin–avidin interactions^66^. Nonetheless, even under controlled *in vitro* conditions, repetitive regeneration using these schemes is challenging and would not be easily adaptable to surface regeneration *in vivo*. eSrtA demonstrates promising utility for the targeted regeneration of molecular constituents *in vivo* that are bound on or potentially within a device. While eSrtA is a bacterial protein and further studies to characterize its immunogenicity are ongoing, precedent does exist for the clinical use of bacterially sourced therapeutics, such as streptokinase, uricase and asparaginase.

In this report, we demonstrate a site-specific covalent immobilization of recombinant TM that has been specifically engineered for enhanced protease stability and oxidation resistance. We observed no decrease in surface-bound TM over 4 weeks *in vitro*; indeed, TM has been characterized with remarkable stability and as such it is a promising candidate for device functionalization. Despite the intrinsic stability of TM, we anticipate the need to regenerate surface activity on permanent blood-contacting prostheses with continuing efforts focused on characterizing the bioactive half-life of surface-bound TM *in vivo.* Importantly, we have determined that eSrtA permits the engineering of covalently bound molecular assemblies onto material surfaces that can be selectively and rapidly ‘recharged' to sustain surface activity. In comparison with other chemoenzymatic approaches^67^,^68^,^69^, the enhanced kinetics and bond-forming efficiency afforded by directed evolution of SrtA was a critical enabling technology for this concept. We confirmed the capacity of eSrtA to catalyse multiple cycles of rapid assembly and removal of LPETG-tagged biomolecules and established that such a strategy could be performed in the presence of whole blood *in vitro* and *in vivo*. Taken together, our findings establish the capacity to rapidly and reproducibly regenerate selective molecular constituents after device implantation with the potential to substantially extend the lifetime of bioactive films and enhance clinically related performance characteristics. It is anticipated that such a strategy could be applied to stripping and regenerating any of a number of potential material bound constituents that display finite stability or activity.

## Methods

### Materials

Unless otherwise specified, all reagents were purchased from Sigma-Aldrich and used without further purification. The peptides NH_2_-GGGGG-N_3_, NH_2_-GGGGGK-biotin, biotin-LPETG and NH_2_-LPETG were obtained from GenScript.

### Synthesis of GGG-PEG5k-biotin

A total of 100 mg of biotin-PEG(5k)-NH2 (Creative PEG works) and 32.3 mg Fmoc-Gly-Gly-Gly-COOH (Santa Cruz Biotechnology) were dissolved in 500 μl anhydrous dichloromethane. A total of 22.3 mg of HBTU and 12.0 mg of hydroxybenzotriazole were dissolved in 1 ml of anhydrous dimethylformamide. The resulting mixture was sonicated for 5 min before the addition of 13.7 μl of N,N-diisopropylethylamine and then reacted overnight with stirring at room temperature. The mixture was quenched on ice by the addition of 10 μl of trifluoroacetic acid and then precipitated by addition to 20 ml of cold diethyl ether. The material was then filtered, dried under reduced pressure and subsequently dissolved in 1 ml of 20% piperidine in dichloromethane and incubated at room temperature for 30 min to remove the Fmoc group. The reaction was quenched by the addition of 0.2 ml trifluoroacetic acid on ice, precipitated with cold ethyl ether and recrystallized twice from cold isopropyl alcohol. The precipitated sample was dissolved in 5 ml of deionized water, dialyzed (MWCO 2 kDa) against deionized water for 24 h, and then lyophilized.

### Alexa Fluor 750-LPETG synthesis

A total of 25 mg Alexa Fluor 750 NHS Ester (Invitrogen) was dissolved in 45 μl of 0.4 M NH_2_-LPETGG peptide in DMSO and incubated at room temperature for 6 h. A total of 2.5 μl DIPEA was then added and incubated at room temperature overnight. Reactions were quenched by the addition of 450 μl of 1 M Tris, pH 7.5, which were incubated on ice for 2 h. The reaction product was purified on a preparative Kromasil 100-5-C18 column (21.2 × 250 mm, Peeke Scientific) by reverse phase HPLC (flow rate: 9.5 ml min^−1^; gradient: 10–70% acetonitrile with 0.1% TFA in 0.1% aqueous TFA gradient over 30 min; retention time 8 min) before pooling and lyophilizing the collected fractions. The concentration of the peptide was determined by the known molar extinction coefficient of Alexa Fluor 750, ɛ_749nm_=290,000 M^−1^ cm^−1^.

### Expression of TM_LPETG_ and sortase variants

Detailed DNA sequence, bacterial expression and purification of TM_LPETG_ and sortase variants are provided in the Supplementary Methods section (Supplementary Table 1). Met-388 was mutated to Leu, which has been previously shown to result in a construct that is more resistant to oxidative inactivation^70^.

### Preparation of fluorescently labelled TM_LPETG_

TM was modified with an amine-reactive fluorescent probe. A total of 4.0 ml of TM_LPETG_ stock in PBS (1 mg ml^−1^) was prepared at 4 °C and 100 μl of Texas Red NHS ester (Invitrogen) at 10 mM in DMSO was added dropwise. The mixture was reacted for 1 h at room temperature with stirring followed by an overnight incubation at 4 °C. The solution was then dialyzed against PBS at 4 °C for 72 h to remove unreacted fluorescent dye and passed through a PD10 column with TBS buffer (25 mM Tris, 150 mM NaCl, pH 7.5). The purified Texas Red-TM conjugate was analysed by UV–vis spectroscopy and SDS–polyacrylamide gel electronphoresis performed by illumination on a standard UV light box.

### Immobilization of TM_LPETG_ on model substrates

Direct sortase-mediated immobilization of TM_LPETG_ on pentaglycine surfaces was tested in streptavidin-coated 96-well microplates (Pierce). A total of 200 μM NH_2_-GGGGG-biotin or biotin-PEG5k-GGG was incubated for 1 h in each well, and washed with TBS (20 mM Tris, 100 mM NaCl, pH 7.5) with 0.05% Tween 20. Various concentrations of TM_LPETG_ were immobilized using 0.1–2 molar equivalents of WT SrtA or eSrtA for 1 h at room temperature. All sortase reactions were performed in a defined reaction buffer consisting of 25 mM Tris-HCl, 150 mM NaCl, 0.5 mM CaCl_2_, pH 7.5. Stripping of immobilized TM was performed using 20 μM SrtA and 1-mM GGG peptide. SrtA-catalysed assembly of TM_LPETG_ was performed in heparinized (20 U ml^−1^) whole blood, which was obtained from healthy volunteers under approval of the BIDMC Institutional Review Board. Whole blood was diluted 50% v/v with normal saline containing SrtA, TM_LPETG_, or GGG with reactions carried out at 37 °C for 1 h without additional calcium. As a reference binding reaction, TM_LPETG_ was biotinylated in a solution containing 100 molar equivalents of NH_2_-GGGGG-biotin and 0.1 molar equivalents of eSrtA for 2 h to generate TM-biotin, which was then incubated in streptavidin-coated microplates for 1 or 16 h. To examine eSrtA-mediated TM_LPETG_ charging and stripping over a 28-day-incubation period, a GGG-functionalized substrate was generated by incubating 200 μl of biotin-PEG5k-GGG for 2 h, followed by a wash in TBS with 0.05% Tween 20. TM_LPETG_ was immobilized using 5 mM TM_LPETG_ and 0.5 mM of eSrtA for 1 h at room temperature in TBS. TM immobilized in 96 wells was washed three times with TBS buffer with 0.5% Tween 20 and then incubated with 200 μl of TBS buffer at 37 °C for 7, 14, and 28 days. At designated time points, sortase-mediated stripping and charging reactions were repeated over three cycles. The quantity of TM immobilized in each well was determined by an ELISA assay per manufacturer's instructions (American Diagnostica). Surface activity of immobilized TM was determined by incubating 100 μl of 0.2 μM human protein C (Calbiochem, Gibbstown), 5 mM calcium chloride and 2 nM human α-thrombin (Haematologic Technologies) in Tris buffer (20 mM Tris, 100 mM NaCl, pH 7.5) with 0.1% bovine serum albumin at 37 °C for 1 h. Activated protein C generation was determined using an enzymatically digestible chromogenic substrate Spectrozyme PCa (Sekisui Diagostics). Experiments from three independent replicates were averaged.

### Modification of catheters *in vitro*

Polyurethane catheters (1 Fr, 2 cm length, SAI Infusion) were derivatized with pentaglycine motifs. For mouse studies, inner and outer catheter surfaces were reacted with 16% v/v hexamethylene diisocyanate (HDI) and 4% v/v triethylamine (TEA) in toluene for 1 h at 50 °C and then rinsed in toluene for 6 h. Isocyanate activated catheters were then reacted overnight at 40 °C with 1 mg ml^−1^ dibenzocyclooctyne (DBCO)-amine linker (Click Chemistry Tools) and TEA (1% v/v) in toluene, rinsed with toluene for 6 h, and dried overnight under vacuum at 25 °C. For rat studies, catheter lumens were heat sealed before surface modification and after isocyanate activation, as described above, catheters were reacted overnight with 2 mg ml^−1^ DBCO-PEG2k-amine linker (Nanocs) and TEA 1% v/v in acetonitrile at 40 °C and then rinsed with acetonitrile for 3 h. To confirm surface cyclooctyne reactivity, DBCO-activated catheters were reacted with 1 mg ml^−1^ tetramethyl-rhodamine-5-carbonyl azide (Invitrogen) in 1:4 tert-butanol/PBS at 37 °C for 24 h followed by rinsing in methanol for 24 h. DBCO activated catheters were reacted overnight with NH_2_-GGGGG-N_3_ at 37 °C and rinsed with TBS buffer. Pentaglycine-modified catheters were incubated with biotin-LPETG or LPETG-labelled Alexa Fluor 750 peptide (2 to 100 μM) and 0.1 molar equivalents of eSrtA relative to the LPETG probe concentration for 30 min or 1 h and rinsed for 4 h in TBS. Biotinylated catheters were incubated in 0.1 mg ml^−1^ Cy3-labelled streptavidin in TBS with 0.05% Tween 20 for 30 min and rinsed overnight in TBS. Imaging of catheters was carried out using the Zeiss Discovery V20 Stereo Microscope and quantitative image analysis by ImageJ was performed to determine fluorescence intensity.

### *In situ* modification of catheters deployed in mice

All animal studies were conducted under the approval of the BIDMC Animal Care and Use Committee. Pentaglycine-modified catheters were deployed ∼1 cm within the inferior vena cava via the femoral vein in 6–8-week-old C57BL/6 mice. All animals were maintained under anaesthesia with isoflurane or ketamine on a heating pad. To functionalize pentaglycine-modified catheters *in situ*, a 200 μl solution containing 4 nmol eSrtA, 40 nmol LPETG-tagged biotin and 20 U of heparin was administered intravenously through the catheter inlet followed by a 100 μl saline flush. To demonstrate *in situ* removal of LPETG-tagged biotin probes from pentaglycine-modified catheters, a 200 μl solution containing 40 nmol eSrtA, 2 μmol GGG peptide and 20 U of heparin was injected intravenously through the catheter inlet followed by a 100 μl saline flush. Mice were sacrificed 1 h later and catheters were explanted and incubated in streptavidin-Cy3 (30 min) and washed with TBS buffer (2 h). Fluorescent and bright field microscopy images were obtained to detect biotin groups on the catheter surface. Real-time imaging of *in situ* removal of LPETG-tagged Alexa Fluor 750 was performed using a Maestro Multi-Spectral *in vivo* fluorescence imaging system with near-IR filter sets. Images were taken using a monochromatic 12-bit camera and analysed using ImageJ to determine fluorescence intensity.

### *In situ* modification of catheters deployed in rats

DBCO-activated PU catheters were generated as described above, reacted overnight with NH_2_-GGGGG-N_3_ at 37 °C, and rinsed in TBS buffer. Free cyclooctyne groups were blocked with 2-azidoethanol (25 mM) in TBS at RT for 1 h and catheters then rinsed with TBS overnight in the dark. Pentaglycine catheters were subsequently reacted with 10 μM Texas Red-TM_LPETG_ and 1 μM eSrtA for 1 h at 37 °C. Under anaesthesia, pentaglycine or Texas Red-TM catheters were deployed within the external jugular vein of Wistar rats (75–100 g, male). The external end of the catheter was trimmed and tucked into the subcutaneous soft tissue and held in place with a suture. Catheters were maintained *in vivo* without administration of a systemic anti-coagulant or anti-platelet agent. Seven days after catheter implantation, rats were anaesthetized and ‘stripping' or ‘charging' reagents were delivered intravenously via the dorsal penile vein (*n*=5/test group). To charge pentaglycine catheters, 80 nmol Texas Red-TM_LPTEG_ with or without 8 nmol eSrtA was delivered (400 μl, saline). To strip catheters, 8 μmol GGG with or without 160 nmol eSrtA was delivered (400 μl, saline). For recharging experiments, catheters were stripped *in vivo* and 24 h later, rats were anaesthetized and charging agents delivered intravenously. Reagents were allowed to circulate for 1 h before catheter removal and imaging by fluorescence microscopy (Axio Zoom, Zeiss). Fluorescent intensity was quantified using Image J64. Catheters displayed no sign of adherent thrombus upon removal (Supplementary Fig. 11).

### Statistical Analysis

Mean and s.d. were calculated for each parameter. Tests for significance between two groups were conducted with the Student's t-test. Tests between three or more groups were conducted with analysis of variance. Values of *P*<0.05 were considered statistically significant.

## Additional information

**How to cite this article:** Ham, H. O. *et al*. *In situ* regeneration of bioactive coatings enabled by an evolved *Staphylococcus aureus* sortase A. *Nat. Commun.* 7:11140 doi: 10.1038/ncomms11140 (2016).

## Supplementary Material

Supplementary Information Supplementary Figures 1-11, Supplementary Table 1 and Supplementary Methods
