# Supplementary material for: In situ regeneration of bioactive coatings enabled by an evolved Staphylococcus aureus sortase A
Source: Nat Commun. 2016 Apr 13;7:11140. doi: 10.1038/ncomms11140 (PMC4833859; doi:10.1038/ncomms11140)
Supplement: Supplementary Information — Supplementary Figures 1-11, Supplementary Table 1 and Supplementary Methods [file ncomms11140-s1.pdf]

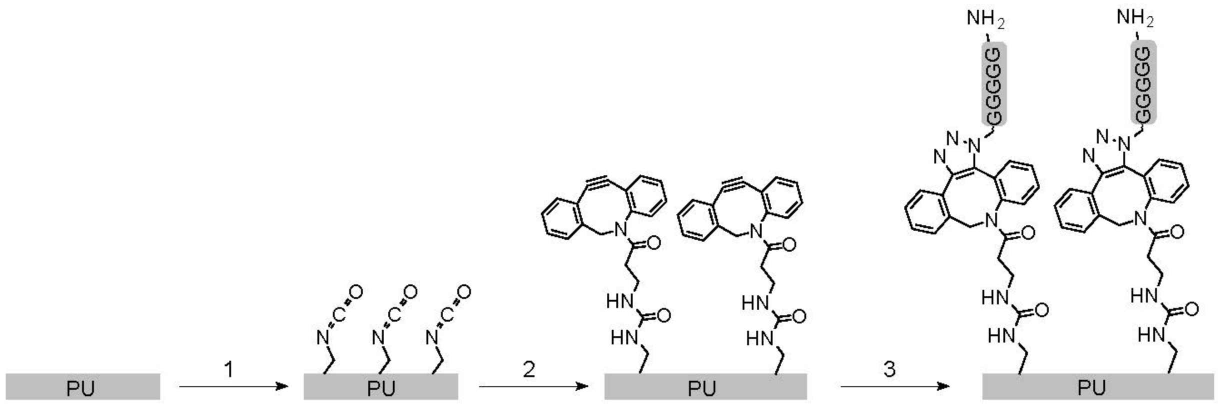

Supplementary Figure 1. Sequential surface reaction scheme to modify polyurethane catheters with pentaglycine (GGGGG) peptide motifs. Reaction conditions: (1) hexamethylene diisocyanate/triethylamine; (2) DBCO-amine/triethylamine; (3) NH_2-GGGGG-N_3.

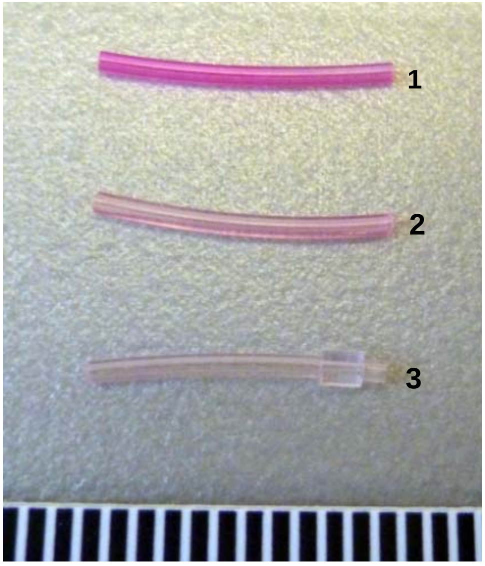

Supplementary Figure 2. Confirmation of dibenzocyclooctyne (DBCO) modification of polyurethane catheters by strain-promoted alkyne-azide cycloaddition. Representative images of samples: (1) DBCO catheter reacted with rhodamine azide; (2) Unmodified polyurethane catheter reacted with rhodamine azide; (3) DBCO modified catheter reacted with rhodamine B.

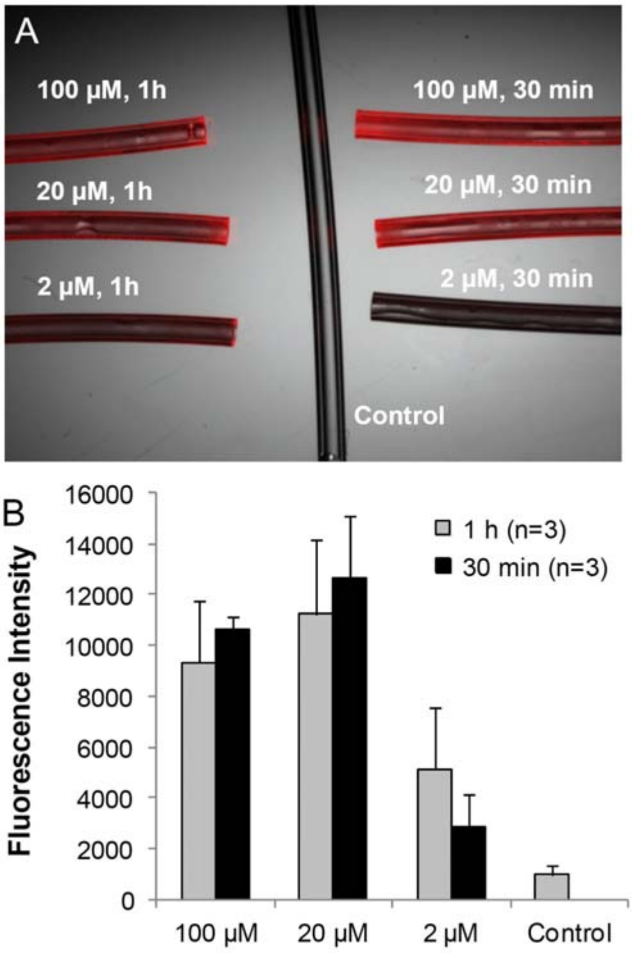

Supplementary Figure 3. Immobilization of LPETG-tagged biotin on pentaglycine modified polyurethane catheters by eSrtA. (**A**) Representative merged fluorescent and bright field microscopy images of polyurethane catheters modified with pentaglycine motifs that were reacted with various concentrations of biotin-LPETG peptide for 30 min or 1 h. A total of 0.1 molar equivalent eSrtA relative to biotin-LPETG was used for all reaction conditions. At the end of the reaction period, catheters were incubated with Cy3-labeled streptavidin at 0.1 $mg \cdot mL^{-1}$ for 30 min to assess the surface density of biotin. (**B**) Fluorescence intensity of biotin-modified catheters after reaction with Cy3-streptavidin measured by Image J. Three individual catheter segments were used and data shown as mean ± standard deviation (n = 3).

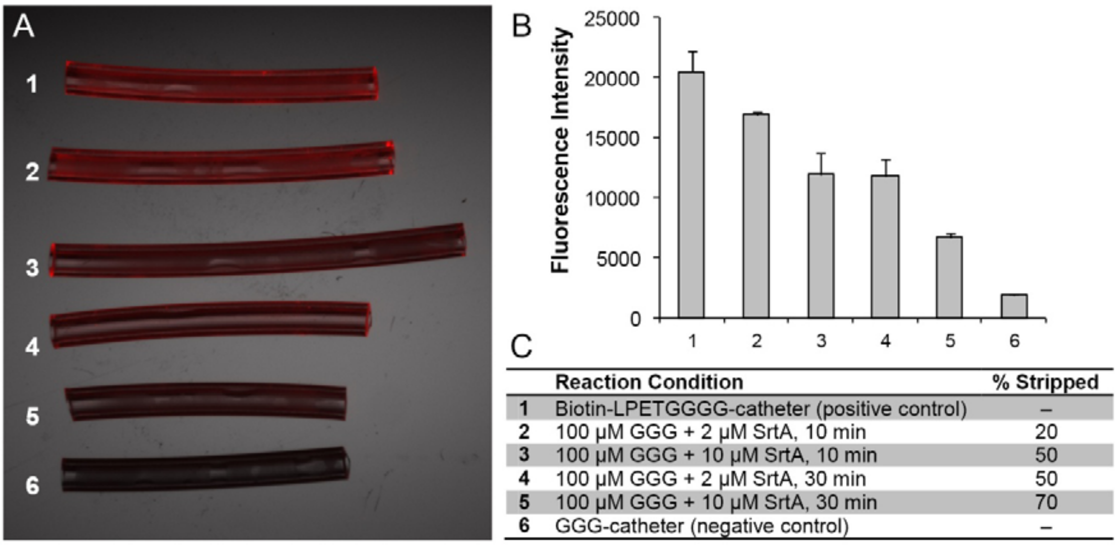

	Reaction Condition	% Stripped
1	Biotin-LPETGGGG-catheter (positive control)	–
2	100 μM GGG + 2 μM SrtA, 10 min	20
3	100 μM GGG + 10 μM SrtA, 10 min	50
4	100 μM GGG + 2 μM SrtA, 30 min	50
5	100 μM GGG + 10 μM SrtA, 30 min	70
6	GGG-catheter (negative control)	–

Supplementary Figure 4. Removal of LPETG-tagged biotin from pentaglycine modified polyurethane catheters via eSrtA stripping reaction. (**A**) Representative merged fluorescent and bright field microscopy images obtained from polyurethane catheters modified with pentaglycine motifs and reacted with biotin-LPETG and eSrtA. Biotin-LPETG was then stripped from catheters using various concentrations of GGG peptide and eSrtA. Catheters were incubated with Cy3-labeled streptavidin at 0.1 $mg{\cdot}mL^{-1}$ for 30 min to assess the surface density of biotin. (**B**) Fluorescence intensity was measured using Image J and expressed as mean ± standard deviation for three individual catheter segments for each reaction condition (n=3). The concentrations of GGG peptide and eSrtA, which were used to strip biotin-LPETG from catheters are summarized in (**C**).

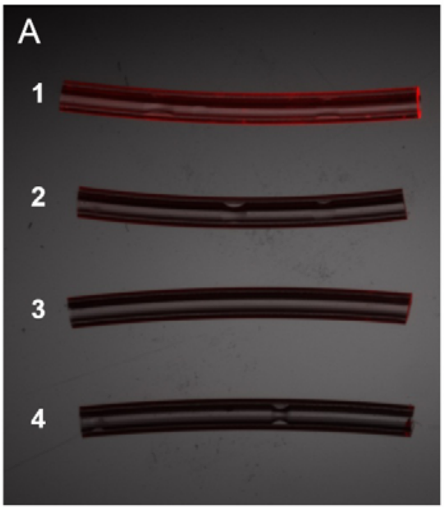

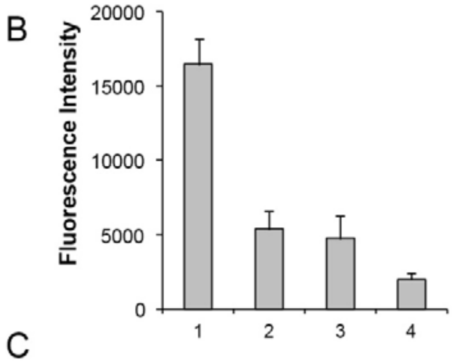

C

	Reaction Condition	% Stripped
**1**	Biotin-LPETGGGG-catheter (positive control)	–
**2**	1 mM GGG + 20 μM SrtA, 10 min	80
**3**	1 mM GGG + 20 μM SrtA, 30 min	80
**4**	GGG-catheter (negative control)	–

Supplementary Figure 5. Removal of LPETG-tagged biotin immobilized on pentaglycine modified polyurethane catheters. (**A**) Representative merged fluorescent and bright field microscopy images of polyurethane catheters modified with pentaglycine motifs and reacted with biotin-LPETG and eSrtA. Biotin-LPETG was then stripped from catheters using various concentrations of GGG peptide and eSrtA. Catheters were incubated with Cy3-labeled streptavidin at 0.1 mg·mL^{-1} for 30 min to assess the surface density of biotin. (**B**) Fluorescence intensity was measured using Image J and expressed as mean ± standard deviation for three individual catheter segments for each reaction condition (n=3). The concentrations of GGG peptide and eSrtA, which were used to strip biotin-LPETG from catheter surfaces are summarized in a Table of reaction conditions (**C**).

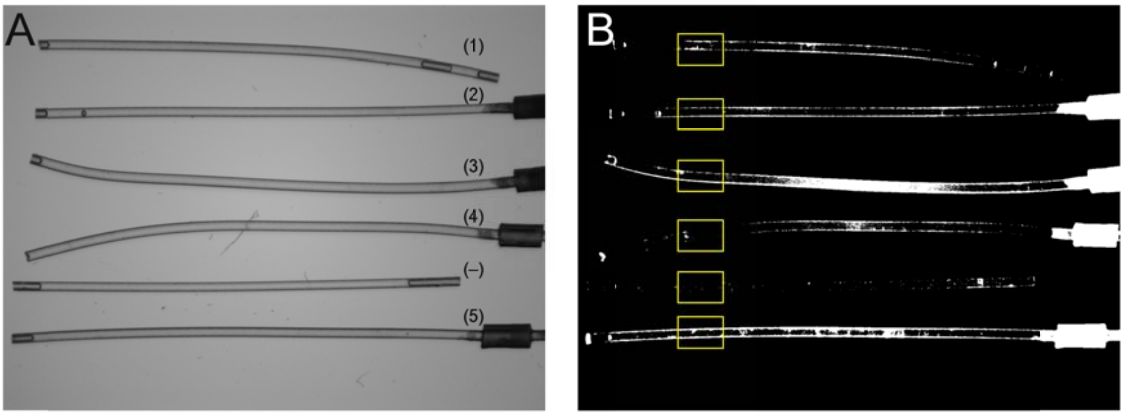

Supplementary Figure 6. ***In situ*** **charging of pentaglycine modified polyurethane catheters deployed in the vena cava of mice with LPETG-tagged biotin.** (**A**) Bright field and (**B**) fluorescent images of catheters, which were deployed in the vena cava of mice and exposed for 1 h to biotin-LPETG peptide (50 µg) and eSrtA (70 µg) administered intravenously. Catheters were explanted and surface charging with biotin-LPETG assessed by streptavidin-Cy3 labeling. Pentaglycine catheters subjected to the *in vivo* charging reaction are labeled **1 – 5** and an unmodified control catheter labeled as (–). Quantitative image analysis of the boxed areas was performed.

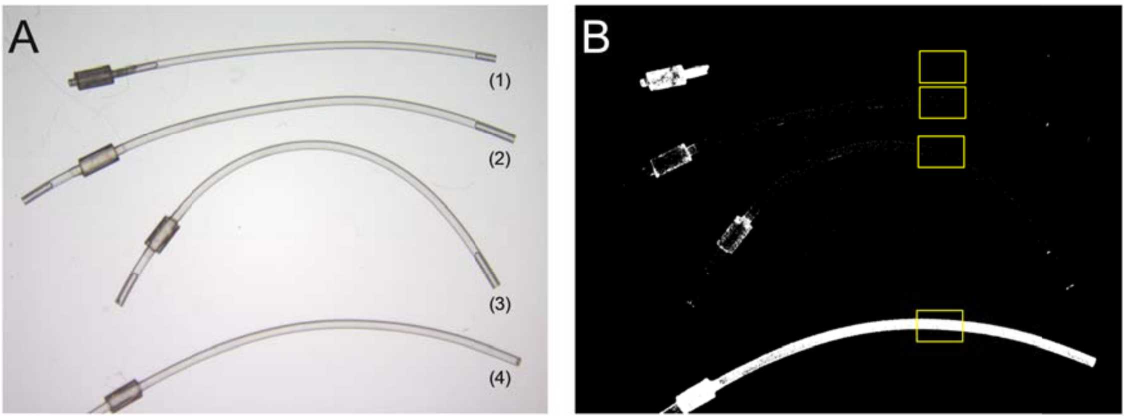

Supplementary Figure 7. Pentaglycine modified polyurethane catheters deployed in the vena cava of mice cannot be charged with LPETG-tagged biotin in the absence of eSrtA. (**A**) Bright field and (**B**) fluorescent images of catheters deployed in the vena cava of mice and and exposed for 1 h to biotin-LPETG peptide (50 μg) administered intravenously. Catheters were explanted and surface charging with biotin-LPETG assessed by streptavidin-Cy3 labeling. Pentaglycine catheter samples subjected to *in vivo* reaction are labeled **1 – 3** and a pentaglycine catheter modified *ex vivo* with biotin-LPETG using eSrtA is labeled as **4**. Quantitative image analysis of the boxed areas was performed.

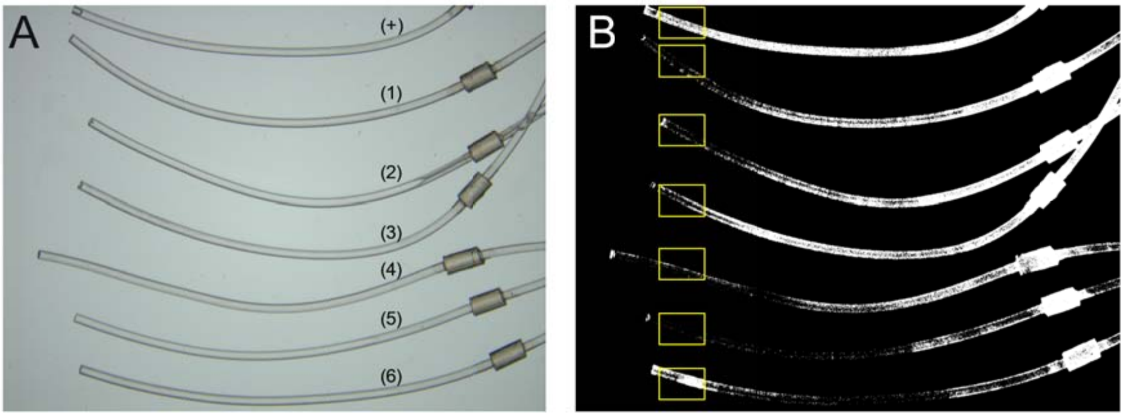

Supplementary Figure 8. ***In situ*** **stripping of LPETG-tagged biotin from pentaglycine modified catheters deployed in the vena cava of mice using eSrtA and triglycine peptide.** (**A**) Bright field and (**B**) fluorescent images of pentaglycine modified catheters deployed in the vena cava of mice and exposed for 1 h to triglycine (400 µg) and eSrtA (700 µg) administered intravenously. Catheters were explanted and surface stripping of biotin-LPETG assessed by streptavidin-Cy3 labeling. Catheters subjected to the *in vivo* stripping are labeled **1 – 6** and a control pentaglycine catheter modified with biotin-LPETG (+). Quantitative image analysis of the boxed areas was performed.

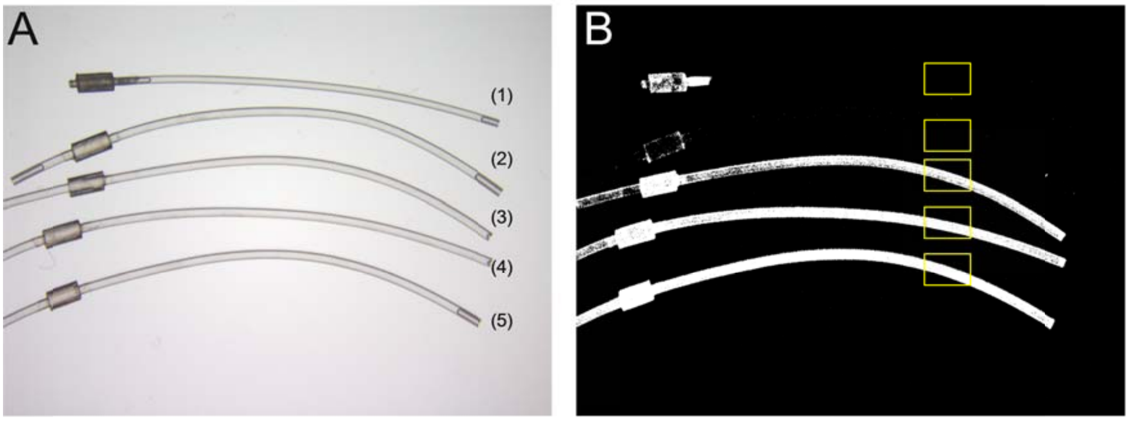

Supplementary Figure 9. ***In situ*** **charging and stripping of pentaglycine modified catheters deployed in the vena cava of mice with LPETG-tagged biotin was not possible in the absence of eSrtA.** (**A**) Bright field and (**B**) fluorescent images of catheters. Pentaglycine modified catheters (**1, 2**) were deployed in the vena cava of mice and exposed for 1 h to biotin-LPETG peptide (50 μg) administered intravenously without associated eSrtA. Pentaglycine modified catheters functionalized *ex vivo* with biotin-LPETG using eSrtA (**3, 4, 5**) were deployed in the vena cava of mice and exposed for 1 h to triglycine peptide (400 μg) administered intravenously without associated eSrtA. Catheters were explanted and the presence of surface bound biotin-LPETG assessed by streptavidin-Cy3 labeling. Fluorescent imaging of streptavidin-Cy3 was performed and signal intensity quantified by image analysis of boxed areas.

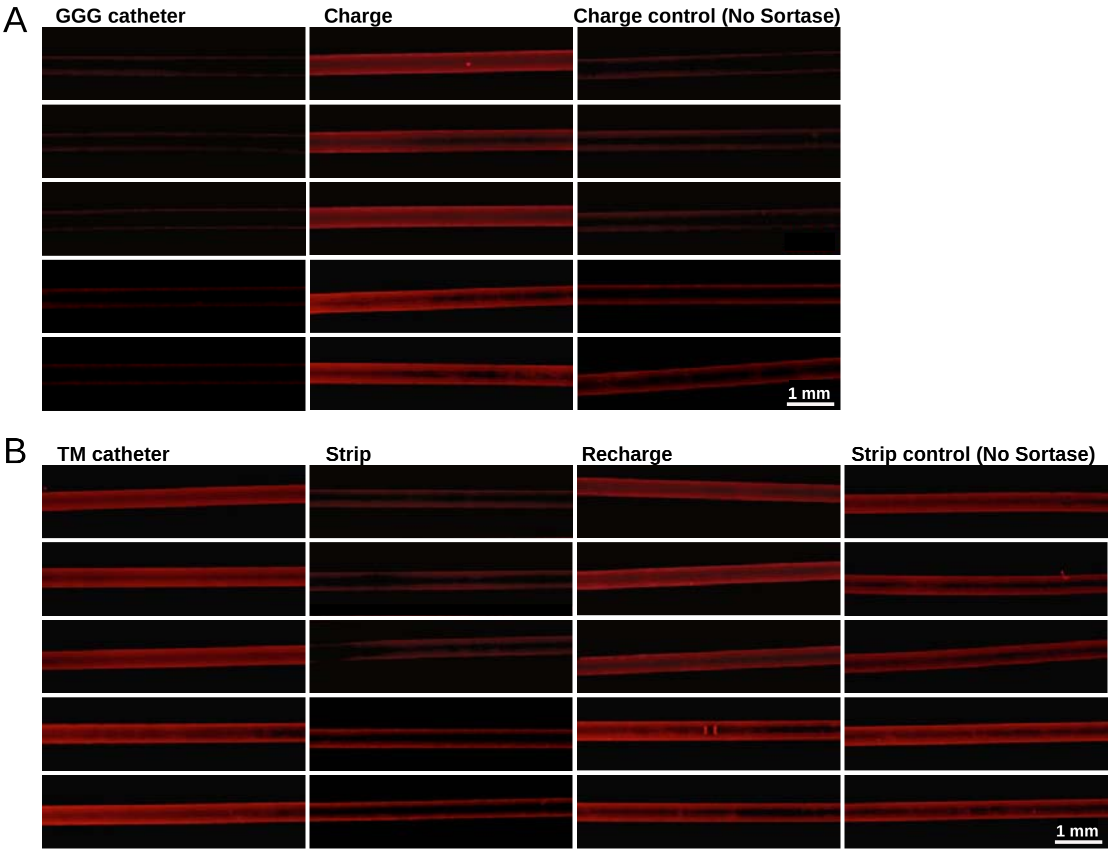

Supplementary Figure 10. Sortase-catalyzed rechargeable assembly of TM_{LPETG} on pentaglycine modified catheters deployed in the rat jugular vein for 7 days. (**A**) To characterize *in situ* charging, pentaglycine modified polyurethane catheters (GGG catheter) were initially deployed in the rat jugular vein for 7 days. Texas Red-labeled TM_{LPETG} with or without eSrtA was delivered intravenously via the dorsal penile vein and 1 h later catheters were explanted and imaged using fluorescent microscopy (n=5). (**B**) To characterize *in situ* stripping, pentaglycine catheters were modified *ex vivo* with Texas Red-labeled TM_{LPETG} (TM catheter) and initially deployed in the rat jugular vein for 7 days. Triglycine with or without eSrtA was then delivered intravenously via the dorsal penile vein and 1 h later catheters were explanted and imaged. To examine *in situ* recharging, TM catheters were stripped *in vivo* after being deployed in the rat jugular vein for 7 days, as described above, and 24 h later, Texas Red-TM_{LPETG} along with eSrtA was administered intravenously via the dorsal penile vein. Catheters were explanted 1 h later for imaging (n=5).

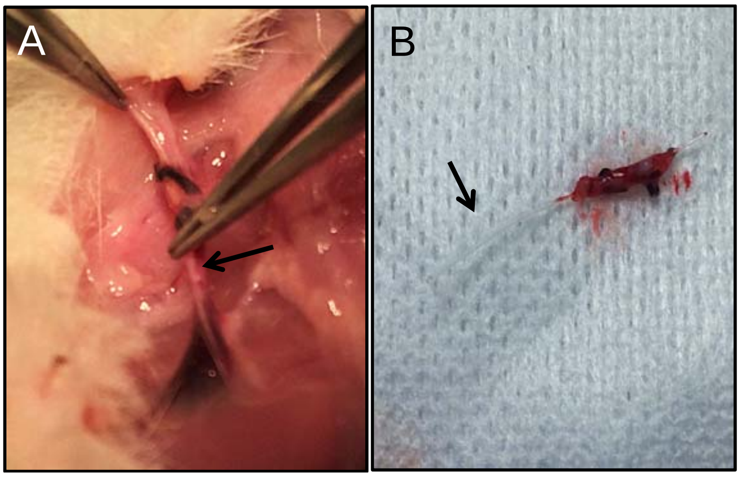

Supplementary Figure 11. Rat model of jugular vein catheterization. (**A**) The ends of a polyurethane catheter (1 Fr, 0.33 mm o.d.) were heat sealed and the catheter deployed in the rat external jugular vein. The proximal end of the catheter was tunneled into the soft tissue of the subcutaneous space in the neck of the rat and sutured in place. (**B**) Representative image of a polyurethane catheter, which was explanted after 7 days in the rat external jugular vein. Catheters were maintained *in vivo* without administration of systemic anticoagulants or anti-platelet agents. Explanted catheters displayed no signs of external thrombus formation.

Supplementary Table 1. Amino acid sequence of the TM_{LPETG}, wild-type sortase (SrtA), and evolved sortase (eSrtA).

Protein	Amino acid sequence
TM_{LPETG} with N-terminal FLAG peptide (15.2 kDa):	MKKTAIAIAVALAGFATVAQADYKDDDDKVKLVEPVDPCFRANC EYQCQPLNQTSYLCVCAEGFAPIPHEPHRCQLFCNQTACPADCDPN TQASCECPEGYILDDGFICTDIDECENGGFCSGVCHNLPGTFECICGP DSALAGQIGTDCGGGGSGGGGSLPETGG
Wild-type sortase with N-terminal His-tag (19.5 kDa):	MASSHHHHHHDYDIPTTENLYFQGSQAKPQIPKDKSKVAGYIEIPDADIKE PVYPGPATPEQLNRGVSFAEENESLDDQNISIAGHTFIDRPNYQFTNLKAA KKGSMVYFKVGNETRKYKMTSIRDVKPTDVGVLDEQKGKDKQLTLITCD DYNEKTGVWEKRKIFVATEVK
Evolved sortase with C-terminal His-tag (17.9 kDa)	MQAKPQIPKDKSKVAGYIEIPDADIKEPVYPGPAT**R**EQLNRGVSFAEENES LDDQNISIAGHTFIDRPNYQFTNLKAAKKGSMVYFKVGNETRKYKMTSIR **N**VKPT**A**VEVLDEQKGKDKQLTLITCDDYNE**E**TGVWE**T**RKIFVATEVKLE HHHHHH

Mutations from the native protein sequence are colored red. OmpA tag sequence is highlighted in blue, which facilitates transport of TM_{LPETG} to the periplasmic space of *E. coli* to optimize folding and is cleaved in final mature TM_{LPETG}. The FLAG peptide sequence of DYKDDDDK at the N-terminus is introduced for protein purification. It has been previously demonstrated that the conversion of the internal methionine residue to leucine maximizes the stability of TM[70]. A second mutation was made to convert the internal RH sequence, which is an active trypsin cleavage site to GQ. The additional sequence at the C-terminus includes a GGGGSGGGGS spacer and the LPETG sortase recognition motif. An extra glycine was added at the C-terminus as this was previously demonstrated to maximize sortase activity.

Supplementary Methods

Expression of TM_{LPETG}. The minimal fragment of human thrombomodulin, epidermal growth factor-like domains 4, 5 and 6 (TM456), was cloned into the Sigma pFLAG ATS expression vector. The sequence is provided in **Supplementary Table 1**. Following transformation of BL21 cells, a fresh LB agar plate was streaked and a single cell colony was then inoculated into 50 mL of media supplemented with 0.4 % glucose and 50 $\mu g \cdot mL^{-1}$ ampicillin and cultured for 16 h at 37°C and 225 RPM. A total of 25 mL of fully grown starter culture (OD600 = 1.20) was then added to 500 mL of media supplemented with 0.4 % glucose and 50 $\mu g \cdot mL^{-1}$ ampicillin, and cultured at 37°C and 225 RPM. Upon cell growth to OD600 = 0.9, IPTG was then added at a final concentration of 1 mM to induce TM_{LPETG} expression and the culture incubated for an additional 4 h at 37°C and 225 RPM. Cell cultures were centrifuged at 4,000x RCF at 4°C for 10 min and stored at 4°C. A standard osmotic shock protocol was performed on stored cell pellets to extract the crude periplasmic proteins. Cell pellets were first warmed to room temperature and re-suspended in 40 $mL \cdot g^{-1}$ cells of 0.5 M sucrose, 0.03 M Tris-HCl (pH 8.0). Suspended cells were evenly distributed into round bottom centrifuge tubes (60 mL per tube). EDTA was then added at a final concentration of 1 mM and cells incubated with gentle shaking for 10 min at room temperature. The cell suspension was centrifuged at 3,500x g for 10 min at 10°C and the supernatant decanted. The cell pellet was rapidly resuspended in 25 mL of ice-cold, distilled water per gm of cell pellet for 10 min and the cell suspension centrifuged at 3,500x RCF for an additional 10 min at 4°C. A total of 35 mL of supernatant was removed from each tube and transferred to clean round bottom centrifuge tubes. These were clarified by further centrifugation at 25,000x RCF for 25 min at 4°C and sterilized using a 0.22 µm filtration system. Anti-FLAG immunoaffinity chromatography (Sigma) was performed on the clarified supernatant per manufacturer's instructions. SDS-PAGE analysis was conducted and total protein quantification was performed using a standard Bradford assay.

Bacterial expression of evolved sortase and wild-type sortase. *E. coli* BL21 transformed with pET29 wild-type sortase or evolved sortase expression plasmids (sequence provided in **Supplementary Table 1**) were cultured at 37°C and 225 RPM in LB media supplemented with 50 $\mu g \cdot mL^{-1}$ kanamycin. Upon OD600 = 0.8, IPTG was added to a final concentration of 0.4 mM and protein expression was induced for 3 h at 30°C. The cells were harvested by centrifugation and resuspended in lysis buffer (50 mM Tris pH 8.0, 300 mM NaCl supplemented with 1 mM $MgCl_2$, 2 $units \cdot mL^{-1}$ DNAseI (NEB), 260 nM aprotinin, 1.2 μM leupeptin, and 1 mM PMSF). Cells were lysed by sonication on ice and the clarified supernatant was purified by column chromatography using Cobalt Talon Resin (Clontech Laboratories) following the manufacturer's instructions. Fractions that were >95 % purity, as judged by SDS-PAGE, were consolidated and dialyzed against Tris-buffered saline (25 mM Tris pH 7.5, 150 mM NaCl) using PD-10 columns (GE Healthcare) and stored as 5 $mg \cdot mL^{-1}$ stocks at 4°C.
